# An effective four-step approach in treating refractory seroma after mastectomy for breast cancer

**DOI:** 10.3389/fonc.2025.1577591

**Published:** 2025-09-10

**Authors:** Rui Cao, Jing Zhang, An Su, Haoshi Bao, Zhou He, Jiannan Wu

**Affiliations:** ^1^ Breast Tumor Center, Sun Yat-Sen University, Guangzhou, China; ^2^ Anesthesiology Department, Sun Yat-Sen Memorial Hospital, Sun Yat-Sen University, Guangzhou, China

**Keywords:** breast cancer, refractory seroma, fibrous capsule, minimally traumatic surgery, flap fixation, local anesthesia

## Abstract

**Background:**

Postoperative chest wall seroma is a common complication following modified radical mastectomy. When persistent, it can lead to the formation of a dense fibrous capsule (pseudobursa), resulting in a refractory seroma that is unresponsive to conventional treatments and may delay essential adjuvant therapies. Surgical excision of the capsule carries significant risks. This study introduces a less invasive surgical technique to manage this challenging condition.

**Methods:**

From 2018 to 2021, 20 patients with refractory seroma after modified radical mastectomy were included in this retrospective study. Inclusion required seroma persistence for over one month despite repeated aspirations, with the presence of a fibrous capsule confirmed by ultrasonography. A minimally traumatic, four-step technique was employed under local anesthesia, involving capsule scraping, “cross-hatch” scoring, and flap fixation. A drainage tube was inserted post-procedure. Color Doppler ultrasound was used for pre- and post-procedural assessment.

**Results:**

The study included 20 female patients with a median age of 57.5 years. All patients had node-positive breast cancer. The “cross-hatch” capsular scoring technique was successfully performed in all cases. The median postoperative drainage time was 7 days (range 6–12 days). During a median follow-up of 3 months, no seroma recurrence was observed. The procedure was well-tolerated with minimal pain, and no significant complications such as hematoma or infection occurred.

**Conclusion:**

The “cross-hatch” capsular scoring technique is a safe, effective, and less invasive method for managing refractory post-mastectomy seroma. This approach minimizes patient trauma, reduces recovery time, and helps maintain the continuity of adjuvant therapies, thereby offering a valuable alternative to more aggressive surgical interventions.

## Introduction

Postoperative chest wall seroma is one of the most common complications after modified radical mastectomy for breast cancer, characterized by the abnormal subcutaneous accumulation of plasma and lymphatic fluid in a dead space (1–3). Its incidence ranges from 15% to 85%, varying by diagnostic criteria and detection methods (4, 5). In some cases, the seroma persists or recurs despite conservative treatments like repeated aspiration or compression, evolving into a refractory seroma. This condition can impede recovery by causing skin necrosis, wound infection, and delays in crucial adjuvant therapies (6), thereby impacting patient outcomes and quality of life.

A key pathological feature of a refractory seroma is the formation of a dense, smooth-walled fibrous capsule, known as a pseudobursa, around the fluid collection (7). This capsule’s avascular, secretory inner lining prevents the adherence of the skin flap to the chest wall, perpetuating the fluid accumulation and rendering conventional treatments ineffective. While complete surgical excision of the capsule (capsulectomy) combined with negative pressure suction has been shown to be effective (8–10), this procedure presents significant challenges. For patients who have undergone axillary lymph node dissection, the capsule often extends into the axilla, in close proximity to vital nerves and vessels. A formal capsulectomy in this region requires extensive dissection, often under general anesthesia, and carries substantial risks of neurovascular injury, intraoperative bleeding, and significant postoperative pain.

Given these risks, there is a clear clinical need for a safer, less invasive alternative. In this study, we developed and evaluated a minimally traumatic surgical technique performed under local anesthesia. The procedure involves scraping and scoring the capsule (“cross-hatch” capsulotomy) without removing it, followed by flap fixation to obliterate the dead space. We herein report our experience, demonstrating the effectiveness and safety of this novel approach in a series of 20 patients.

## Patients and methods

### Patient selection

Between 2018 and 2021, 20 consecutive female patients with refractory seroma following modified radical mastectomy at our institution were included in this retrospective study. The inclusion criteria were: 1) persistence of seroma for over one month despite conventional management (including repeated percutaneous aspirations); and 2) the presence of a distinct, organized fibrous capsule confirmed by preoperative ultrasound examination. All patients had been pathologically diagnosed with breast cancer and had undergone axillary lymph node dissection as part of their initial surgery. Of these 20 patients, 10 underwent a standard Level I and II axillary lymph node dissection, while the remaining 10, who presented with clinically enlarged Level III lymph nodes, underwent a complete Level I-III dissection. The refractory seromas in this cohort developed after the removal of the initial surgical drains, which were typically kept in place for a period of 7 to 14 days.

The choice of modified radical mastectomy (MRM) for the initial surgery was based on clinical indications at the time of diagnosis, such as multicentric disease, large tumor-to-breast ratio, or patient preference, reflecting the clinical practice at our institution during the study period. Patients were excluded if they had an active infection, severe coagulation disorders, or declined the procedure. Since this study was conducted retrospectively based on a modification of an established clinical technique, formal ethical committee approval was not required by our institutional guidelines.

### The cross-hatch capsular scoring technique

**Figure 1:**
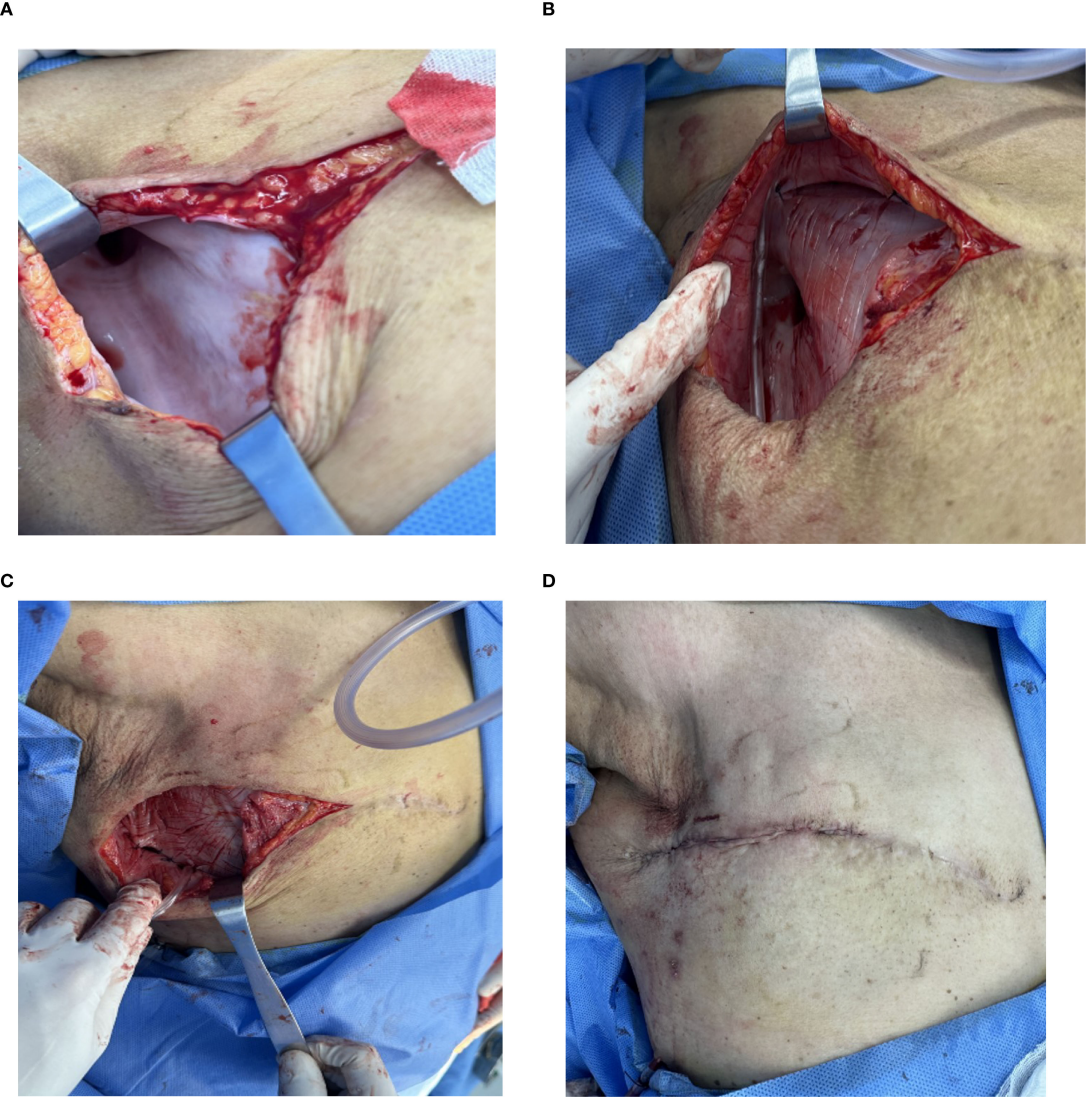
Key Steps of the Minimally Traumatic Surgical Procedure. **(A)** Exposure of the Pseudobursa: Following incision and complete fluid aspiration, the characteristic smooth, white fibrous capsule is visualized covering the chest wall and extending into the axilla. **(B)** Capsular Scoring: A scalpel is used to create multiple shallow, crisscrossing incisions (“cross-hatch” pattern) across the entire inner surface of the capsule, promoting an inflammatory healing response. The capsule itself is preserved. **(C)** Flap Fixation and Drainage: To ensure apposition, the skin flap is sutured to the underlying muscle fascia using 2–0 absorbable sutures. A drainage tube is placed to manage initial exudate. **(D)** Wound Closure: The incision is re-approximated with absorbable sutures, completing the procedure before compression bandaging is applied.

The surgical procedure was performed in an outpatient setting and comprises four essential steps (**Figure 1**).

1. Incision and Exposure: After routine ultrasonography to map the extent of the seroma, the area was sterilized. Using 0.5% lidocaine for local anesthesia, an incision of 7–10 cm was made along the original mastectomy scar to access the seroma cavity. The seroma fluid was completely aspirated with a suction device, revealing the dense, smooth-walled fibrous capsule (pseudobursa) lining the cavity (**Figure 1A**). The aspirated fluid was visually inspected; as all cases presented with clear, serous fluid without signs of infection (e.g., purulence or turbidity), routine microbiological cultures were not performed.
2. Capsular Scoring: The key step involves altering the capsule surface. Using a No. 22 scalpel blade, the entire inner surface of the capsule on the skin flap and chest wall was methodically scraped to de-epithelialize the secretory lining. Following this, multiple “cross-hatch” scores or shallow incisions were made through the capsule down to the subcutaneous tissue or muscle layer (**Figure 1B**). Care was taken in the axillary region to avoid deep incisions that could injure the axillary vein or surrounding nerves. The fibrous capsule itself was left *in situ*.
3. Flap Fixation and Drainage: To obliterate the dead space, the skin flap was anchored to the underlying pectoral or serratus anterior muscle fascia using multiple interrupted 2–0 absorbable sutures (**Figure 1C**). A single closed-suction drainage tube was then placed within the cavity.
4. Closure and Compression: The skin incision was closed in layers. A sterile dressing and a multi-layer elastic compression bandage were applied to the chest wall to ensure firm apposition of the flaps (**Figure 1D**). The drain was connected to a low-pressure suction bottle. A single dose of a first-generation cephalosporin was administered preoperatively for surgical prophylaxis in all cases.

### Post-procedure management and follow-up

The drainage tube was removed when the daily output was consistently less than 20 ml for two consecutive days. The compression dressing remained in place until after drain removal. All patients underwent a color Doppler ultrasound examination one week after drain removal to confirm the absence of fluid re-accumulation. Follow-up was conducted at 1 and 3 months post-procedure to assess for seroma recurrence and any late complications.

## Results

### Patient and tumor characteristics

A total of 20 female breast cancer patients with refractory seroma were included. The median age was 57.5 years (range: 47-78), and the mean Body Mass Index (BMI) was 24.6 kg/m ² (range: 19.4-35.5 kg/m ²). Comorbidities included diabetes mellitus in 4 patients (20%) and hypertension in 8 patients (35%). Six patients (30%) had received neoadjuvant chemotherapy.

Postoperative histopathology from the initial mastectomy revealed stage IIB in 8 patients (40%), IIIA in 6 patients (30%), and IIIC in 6 patients (30%). The median number of total lymph nodes removed was 17 (range: 7-30). All 20 patients had positive lymph nodes, with a median of 3 affected nodes (range: 1-25). Patient characteristics are summarized in **Table 1**.

**Table 1:** Clinicopathlogical characteristics of the patients.

Item	Results
Median age	57.5 yrs (rang 47-78 yrs)
Body mass index (Mean standard deviation, kg/m^2^)	24.6 (±3.98)
Hypertension (Cases)	8/20
Neoadjuvant chemotherapy	6/20
adjuvant chemotherapy	14/20
Diabetes (Cases)	4/20
Number of dissected lymph nodes	
<10	1
10-20	11
>=20	9
Number of metastatic lymph nodes	
<4	7
4-9	7
>=10	6
Post opertive Stage	
2B	8
3A	6
3C	6

### Procedural outcomes

The “cross-hatch” capsular scoring technique was successfully performed on all 20 patients under local anesthesia.

The median postoperative drainage time was 7 days (range 6–12 days). No major perioperative complications such as hematoma, surgical site infection, or flap necrosis were observed. Patients reported minimal postoperative pain, which was well-managed with oral analgesics.

During a median follow-up of 3 months, no recurrence of seroma was observed in any patient. Post-procedural ultrasound examinations confirmed complete resolution of the fluid collection and adherence of the skin flaps to the chest wall.

## Discussion

Managing postoperative refractory seroma in breast cancer surgery remains a significant clinical challenge. Our study demonstrates that the “cross-hatch” capsular scoring technique is a highly effective and safe method for treating this condition.

The rationale behind our technique is not merely to drain fluid but to fundamentally alter the pathophysiology of the non-healing seroma cavity. The pseudobursa’s smooth, avascular inner lining prevents natural tissue adherence and perpetuates fluid secretion. By mechanically scraping and scoring this surface, we create a controlled inflammatory response. This, combined with meticulous flap fixation and external compression, promotes fibrin deposition and granulation, leading to the permanent obliteration of the dead space. The temporary drain serves only to manage the initial reactive exudate while this crucial adherence process occurs. Our results, showing no recurrences in 20 consecutive patients, provide strong preliminary evidence that this method offers a durable solution.

The standard surgical alternative for an encapsulated seroma is a formal capsulectomy. Unlike this procedure, which requires extensive and often high-risk dissection under general anesthesia, our technique is minimally traumatic. By leaving the capsule’s outer layer intact and avoiding deep dissection, especially in the axilla, we minimize the risk of bleeding and neurovascular injury. Other innovative approaches, such as ultrasound-guided scraping with Negative Pressure Wound Therapy (NPWT), have been described (11). Our method, however, offers distinct advantages in its simplicity and accessibility, as it utilizes standard surgical instruments and does not require specialized equipment. The limited incision allows for direct visualization, ensuring thorough treatment of the capsule surface while protecting vital structures.

A key strength of our approach is its performance under local anesthesia. This makes it suitable for a wider range of patients, including the elderly or those with comorbidities who may be poor candidates for general anesthesia. The minimal postoperative pain reported by our patients, combined with the outpatient nature of the procedure, enhances patient comfort, reduces healthcare costs, and lessens the psychological burden associated with recurrent clinic visits.

This study has several limitations. Firstly, it is a retrospective, single-arm study with a relatively small sample size. Therefore, it should be considered a proof-of-concept series demonstrating the technique’s feasibility and initial efficacy. Secondly, the follow-up period is limited to three months, although this is generally sufficient to detect early seroma recurrence. Future research should focus on larger, prospective, and potentially comparative studies to further validate these findings and assess long-term outcomes.

## Conclusion

The “cross-hatch” capsular scoring technique represents a significant advancement in the management of postoperative refractory seroma. Its minimally traumatic nature, high efficacy, and excellent safety profile make it a valuable option for surgeons and patients alike. By adopting this method, clinicians can effectively resolve a challenging postoperative complication, improve recovery times, reduce morbidity, and ensure the timely continuation of essential adjuvant therapies, ultimately enhancing the quality of care in breast cancer surgery.

## Data Availability

The original contributions presented in the study are included in the article/Supplementary Material. Further inquiries can be directed to the corresponding author.
